# Morphological and histochemical characterization of eyelids and orbital glands in the crocodile monitor (*Varanus salvadorii*)

**DOI:** 10.1038/s41598-026-57505-2

**Published:** 2026-06-11

**Authors:** Joanna Klećkowska-Nawrot, Aleksander Chrószcz, Shaw Badenhorst, Konrad Cyprych, Abit Aktaş, Wojciech Paszta, Karolina Goździewska-Harłajczuk, Dominik Poradowski

**Affiliations:** 1 Department of Biostructure and Animal Physiology, Division of Animal Anatomy, Faculty of Veterinary Medicine, Wrocław University of Environmental and Life Sciences, Kożuchowska 1, Wrocław, 51-631 Poland; 2 Evolutionary Studies Institute, University of the Witwatersrand, 1 Jan Smuts Ave, Braamfontein, Johannesburg, 2000 South Africa; 3 Soft Matter Optics Group – SMOG, Faculty of Chemistry, Wrocław University of Science and Technology, Wybrzeże Wyspiańskiego 27, Wrocław, 50-370 Poland; 4 Department of Histology and Embryology, Faculty of Veterinary Medicine, Istanbul University-Cerrahpasa, Istanbul, Turkey; 5 Wrocław Zoological Garden, Z. Wróblewskiego 1-5, Wrocław, 51-618 Poland

**Keywords:** Crocodile monitor, Harderian gland, Lacrimal gland, Lower eyelid, Nictitating membrane, Upper eyelid, Anatomy, Diseases, Medical research, Physiology, Zoology

## Abstract

The crocodile monitor is an arboreal predator, and its ocular adnexa remain poorly characterized. This study provides the first detailed histochemical characterization of the ocular adnexa in this species, including the upper and lower eyelids, the nictitating membrane, the Harderian gland, and the lacrimal gland, based on two adult individuals. Because the available material was limited, the findings should be interpreted as descriptive baseline observations. Histological slides were analyzed using microanatomical assessment, histochemistry, and Picrosirius Red staining under polarized light. Both eyelids were covered by scales and contained a pigmented corneous β-protein–rich epidermis. A collagen-rich dermis with a thick stratum compactum and an osteoderm located between the dermis and hypodermis were identified. The posterior palpebral epithelium was non-keratinized and contained abundant goblet cells producing mainly acidic mucins. The lower eyelid also contained a dense regular tarsal plate and lymphoid tissue. The nictitating membrane was folded and pigmented, contained hyaline cartilage, and showed conjunctiva-associated lymphoid tissue. Birefringence patterns suggested Type I–like collagen in the perichondrium and Type III–like collagen in the cartilage matrix. The Harderian gland, a large acinar gland, opened onto the bulbar surface of the nictitating membrane and secreted mainly acidic mucins with a moderate neutral mucin component. The small lacrimal gland produced predominantly acidic mucins without detectable neutral mucins. These findings provide a baseline for the protective and secretory ocular structures of the crocodile monitor and represent the first detailed histochemical characterization of the studied orbital glands. In addition, this study contributes to comparative anatomy, veterinary medicine, and exotic animal conservation.

**Supplementary Information:**

The online version contains supplementary material available at 10.1038/s41598-026-57505-2.

## Introduction

The crocodile monitor, also known as the Papuan monitor or Salvadori’s monitor (*Varanus salvadorii*), was first described by Peters and Doria in 1878. The species was later transferred to the genus *Varanus*^1^. Based on selected anatomical traits, Mertens^2^ proposed a monotypic subgenus, *Papusaurus*, within *Varanus*. Subsequent morphological analyses (including hemipenial and lung morphology), as well as biochemical and molecular studies, indicated the distinct position of the crocodile monitor within monitor lizards and its affiliation with the Indo-Australian lineage^1^. More recent DNA-based phylogenetic analyses (three nuclear genes and two mitochondrial genes) classified the crocodile monitor within a clade that also includes *V. varius* and *V. komodoensis*^3^. The occurrence and ecology of the crocodile monitor remain poorly known, most likely because of its strongly arboreal lifestyle and preference for remote, difficult-to-access primary forest habitats^1–4^. This species is endemic to New Guinea (including Salawati Island), and its habitats are associated with various types of rainforest, including alluvial, montane, and riparian forests. It has also been recorded in mangroves and in the mosaic of more open environments of the Trans-Fly region^4–6^. It is a diurnal, arboreal species observed in dense forest vegetation and along rivers^7^.

In view of the crocodile monitor’s biology, including life in a humid, densely vegetated environment and frequent activity within the forest canopy, adaptations of protective ocular structures are likely to play a crucial role. Therefore, the ocular adnexa, such as the eyelids and orbital glands, are of particular interest, and a complete morphological and histochemical study may help fill an important gap in the literature.

In reptiles, the eyelids and orbital glands constitute a single functional system that mechanically protects the eyeball and helps maintain proper ocular surface homeostasis through the palpebral conjunctiva and secretions produced by the Harderian gland and lacrimal gland^8^. In most lizards, both the upper and lower eyelids are present, and goblet cells occur on the posterior palpebral surface, which is associated with the production of mucous components of the tear film^8,9^. However, within Squamata, this system can be profoundly modified. In some species, the eyelids fuse and form a transparent covering (“spectacle”), which typically co-occurs with the reduction or absence of the nictitating membrane. Some species lack the lacrimal gland^10–13^. In such arrangements, the Harderian gland, which is widespread in reptiles, is of particular importance. This organ shows morphological variability and may perform functions beyond simple lubrication of the ocular surface, depending on the taxon^14–17^. Moreover, interspecific comparisons indicate that even pronounced differences in orbital anatomy or lifestyle do not necessarily translate into obvious remodeling of the Harderian gland itself (e.g., in *Lerista* skinks). These observations highlight the complexity of the relationship between morphology and secretory function^18,19^. At the same time, the conjunctival surface of the eyelids is relevant not only for secretion but also for immunity. The presence of CALT (conjunctiva-associated lymphoid tissue) indicates the involvement of the conjunctiva in local immune mechanisms^20–24^.

The aim of this study was to provide the first complete morphological, histological, and histochemical description of the eyelids and ocular glands in the crocodile monitor. These data allow the identification of features that may be functionally important in ocular surface protection. The study material was obtained from two individuals, a male and a female, that had lived at the Wrocław Zoological Garden (Poland), and tissues were collected for analysis after death. Therefore, the findings may serve as a reference point for interspecific comparisons and animal care in zoological gardens. The study also provides a basis for further comparative studies of material from wild-living individuals.

## Materials and methods

### Specimen collection and anatomical data acquisition

**Fig. 1:**
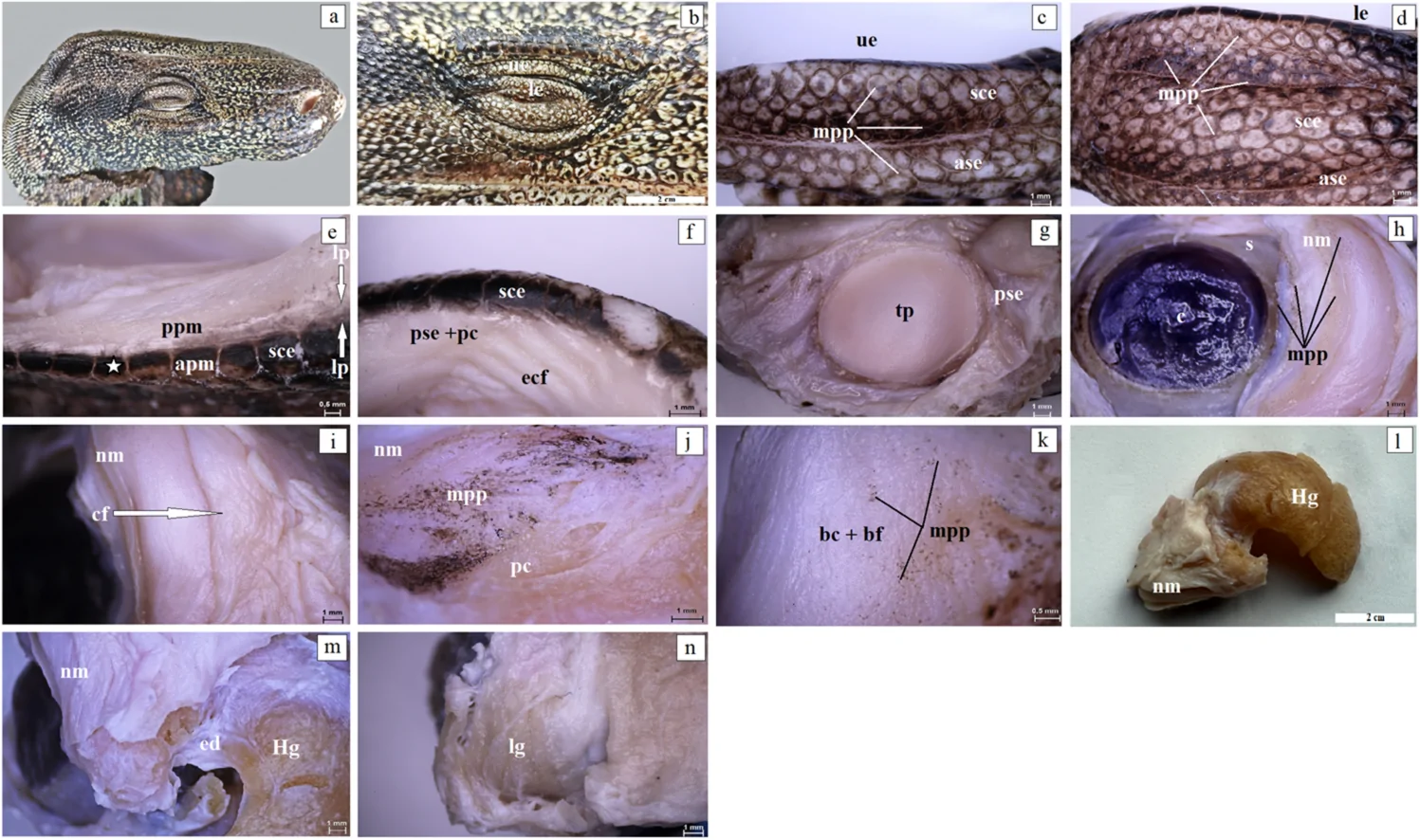
Anatomy of the eyelids, nictitating membrane, and associated glands in the crocodile monitor. (**a**) – Head. (**b**) – Upper (ue) and lower eyelids (le). (**c**,**d**) – Anterior eyelid surfaces (ase) showing eyelid scales (sce) and melanophore pigmentation (mpp). (**e**) – Anterior and posterior palpebral margins (apm, ppm); the white asterisk indicates melanophore pigmentation of the anterior palpebral margin; two lacrimal puncta (lp) are indicated by the white arrow. (**f**) – Posterior eyelid surface (pse), palpebral conjunctiva (pc), and eyelid conjunctival folds (ecf). (**g**) – Posterior surface of the lower eyelid (pse) and tarsal plate (tp). (**h**) – Nictitating membrane (nm) in relation to the cornea (c) and sclera (s); mpp is indicated. (**i**) – Nictitating membrane (nm) and conjunctival folds (cf.). (**j**) – Nictitating membrane (nm) and palpebral conjunctiva (pc) with mpp. (**k**) – Bulbar conjunctiva (bc) and bulbar folds (bf) with mpp. (**l**) – Harderian gland (Hg) and nictitating membrane (nm). (**m**) – Excretory duct (ed) of the Harderian gland (Hg) and nictitating membrane (nm). n – Lacrimal gland (lg). Scale bars: (**b**, **l**) 2 cm; (**c**, **d**, **f**–**j**, **m**, **n**) 1 mm; (**e**, **k**) 0.5 mm.

Two adult crocodile monitors from the Wrocław Zoological Garden (Poland) were examined. The specimens were collected post-mortem between 2020 and 2023 (Fig. 1a). At the time of natural death, the female was 11 years, 11 months, and 15 days old, and the male was 9 years, 7 months, and 20 days old. Body mass was not recorded at the time of death. Neither individual was euthanized for this study. The animals lived in a standard zoological environment with routine veterinary care according to the Wrocław Zoological Garden protocols. Although the crocodile monitors had access to outdoor enclosures, no ocular abnormalities were recorded during intravital routine clinical veterinary examinations or post-mortem examination. Anatomical dissection was carried out in the Division of Animal Anatomy, Department of Biostructure and Animal Physiology, and in the Natural History Museum of the Faculty of Biology and Animal Sciences, Wrocław University of Environmental and Life Sciences.

According to its current conservation status, the crocodile monitor is classified as a species of Least Concern (LC) according to the IUCN Red List of Threatened Species (2025-2).

Immediately after death, photographic documentation of the head, upper eyelid, lower eyelid, nictitating membrane and orbital glands was performed using a Nikon D300s camera with a Tamron AF 17–50 mm F/2.8 [IF] lens (Nikon Corporation, Tokyo, Japan). Pre-fixation measurements and tissue sampling were completed within 2 h *post-mortem*. Morphometry of the studied anatomical structures was performed prior to fixation, before the specimens were placed in 4% buffered formaldehyde, to avoid fixation-related dimensional changes, in accordance with the adopted anatomical protocol. Morphometric measurements of the upper and lower eyelids (length, width, and thickness; six repeated measurements each) were taken on both body sides using a digital slide caliper with an accuracy of ± 0.02 mm (Handy Worth, Poland).

The next step involved dissection of the upper and lower eyelids in both individuals to collect the entire eyeballs along with the nictitating membrane, Harderian gland, and lacrimal gland. The study material included the upper eyelid (*n* = 4), lower eyelid (*n* = 4), nictitating membrane (*n* = 4), Harderian gland (*n* = 4), and lacrimal gland (*n* = 4). Macroscopic features that were not readily visible with the naked eye were examined using a Zeiss Stemi 2000-C stereomicroscope (Carl Zeiss, Jena, Germany), with photographic documentation captured using a Canon EOS 300D camera (Canon Inc., Tokyo, Japan). For each morphometric feature of the eyelids and orbital glands, a representative value was calculated as the bilateral mean value. The standard deviation (SD) was also calculated. Because of the limited amount of available material, statistical analysis was substantially restricted. The results are descriptive only, and no inferential statistics were employed. Thus, most of the obtained data are descriptive in character and serve only for preliminary characterization of the studied structures.

### Tissue processing and paraffin embedding

All tissue samples were fixed after collection to preserve tissue architecture and allow consistent and comparable histological and histochemical analyses. The tissue samples were fixed in 4% buffered formaldehyde for at least 72 h and then rinsed in running water for 24 h. Dehydration was performed using a graded ethanol series in a vacuum tissue processor (ETP), followed by paraffin embedding. Tissue Sect.  4 μm thick were obtained using a sliding microtome. Microscopic imaging was conducted using a light microscope equipped with dedicated software. Histochemical analysis was aimed at assessing the composition of secretory products of goblet cells on the posterior surfaces of the upper and lower eyelids, as well as in the bulbar and palpebral conjunctiva of the nictitating membrane, and at characterizing the secretion of the orbital glands (Harderian gland and lacrimal gland). Histochemical techniques were applied according to the methodology described by Spicer and Henson^25^. All detailed protocol data are available in Supplementary File S1. The terminology used in the histological descriptions was based on the *Nomina Histologica Veterinaria*^26^.

### Histological and histochemical stains

Histological and histochemical analyses were performed using a comprehensive panel of classical and specialized staining methods for detailed analysis of the structure and secretory nature of the accessory organs of the eye in crocodile monitors. For general structural characterization and connective tissue analysis, the following stains were used: Mayer’s hematoxylin and eosin^27^, Heidenhain’s Azan trichrome, Masson–Goldner trichrome, Mallory trichrome with aniline blue, Reticulin, and Verhoeff. For mucin secretion detection, Movat pentachrome (modified Russell–Movat) and Mucicarmine were applied. For identification of specialized components, Bielschowsky, Masson–Fontana, and toluidine blue stains were used. Histochemical analysis was used to characterize glycans and mucosubstances. All details are available in Supplementary File S1.

### Terminology notes

In this study, proteins historically referred to as β-keratins are described as corneous β-proteins (CBPs) to avoid confusion with true keratins (intermediate filament keratins). Accordingly, the “outer” and “inner β-keratin layers” described in the histological figures correspond to outer and inner CBP-rich layers, respectively, whereas α-keratin refers to intermediate filament keratins^28–30^.

### Polarized microscopy analysis

To determine the type and organization of collagen fibers (Types I and III–like collagen) in the upper eyelid, lower eyelid, nictitating membrane, and Harderian gland of the crocodile monitor, histological sections were stained with Picrosirius Red^31^. The stained sections were examined under a polarized-light microscope to differentiate collagen types^32^. Collagen type was inferred indirectly from birefringence patterns and color, which are influenced by fiber thickness, packing, and section orientation. This approach allowed assessment of collagen network distribution and fiber orientation in the upper eyelid, lower eyelid, and nictitating membrane of the crocodile monitor. Imaging was performed to capture the full structural layout of the eyelids. Representative regions were selected to highlight specific collagen morphology. The interaction of Picrosirius Red with collagen fibers causes rotation of the light polarization vector, visible under crossed polarizers.

### Ethical statement

The study was carried out according to applicable Polish law (Journal of Laws of January 15, 2015 – Act on the Protection of Animals Used for Scientific or Educational Purposes) and European regulations (Directive 2010/63/EU of the European Parliament and of the Council of September 22, 2010, on the protection of animals used for scientific purposes). According to both legal acts, studies involving only post-mortem material from the Wrocław Zoological Garden do not require approval from the Ethics Committee. The animals were obtained under individual permits (No. PIW Wroc. UT-45/5/16 – Dr. Joanna Klećkowska-Nawrot; No. PIW Wroc. UT-45/6/16 – Dr. Karolina Goździewska-Harłajczuk) issued by the District Veterinary Officer in Wrocław (Poland).

## Results

### The upper and the lower eyelids

**Fig. 2:**
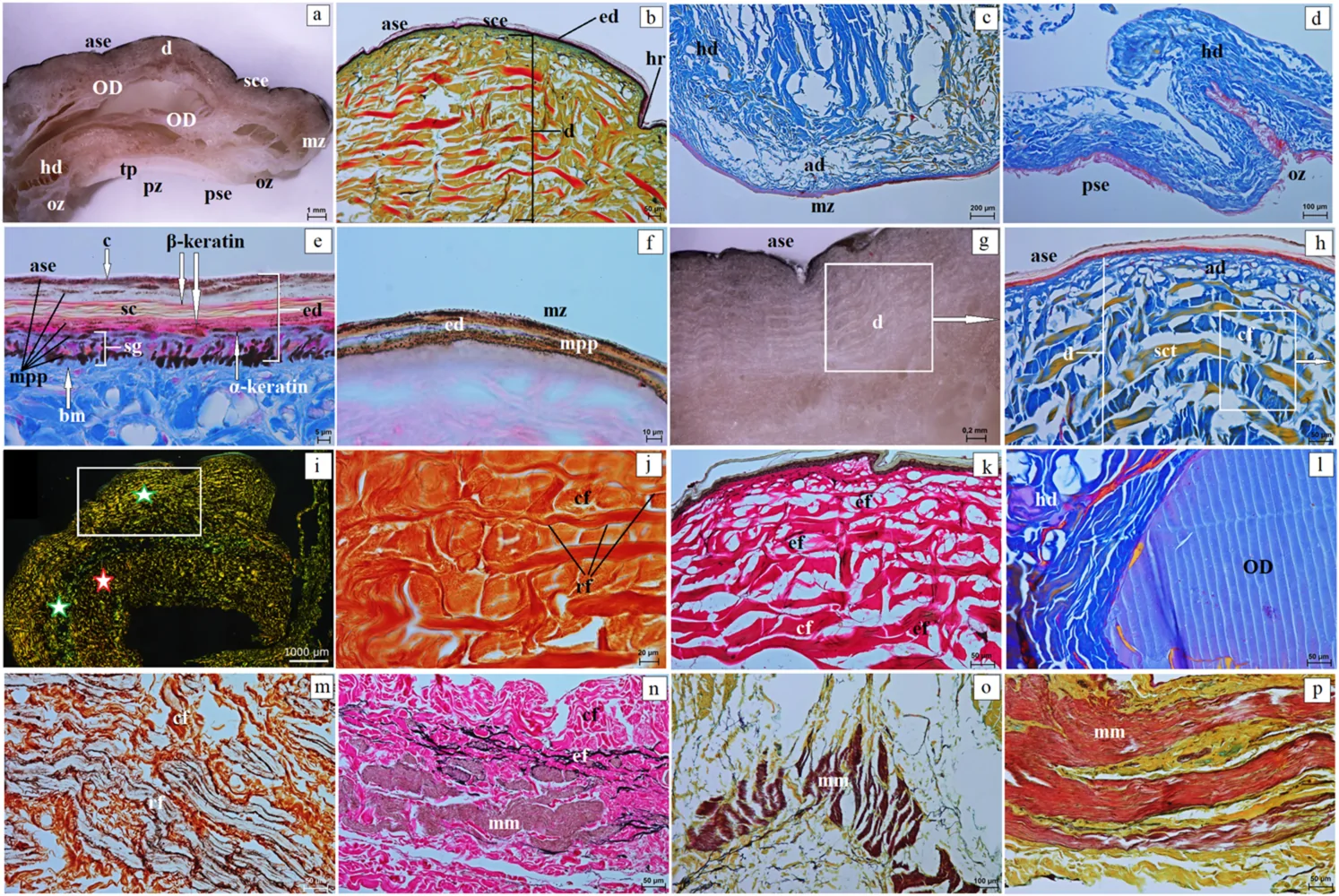
Histology of the upper and lower eyelids in the crocodile monitor. Adipocytes (ad), anterior surface of eyelid (ase), basement membrane (bm), collagen fibers (cf.), cuticle (c), dermis (d), epidermis (ed), elastic fibers (ef), hypodermis (hd), hinge region (hr), muscles (mm), melanophore pigmentation (mpp), marginal zone (mz), osteoderm (OD), ocular zone (oz), posterior surface of eyelid (pse), plate zone (pz), reticular fibers (rf), stratum corneum (sc), scale (sce), stratum compactum (sct), stratum germinativum (sg), tarsal plate (tp); green asterisk indicates Type III–like collagen (green–yellow birefringence), red asterisk indicates Type I–like collagen (orange–yellow birefringence). (**a**,**g**) – Stereomicroscope; (**c**–**e**,**h**,**l**) – Picro-Mallory trichrome; (**f**) – Masson–Fontana; (**b**,**o**,**p**) – Movat pentachrome (modified Russell–Movat); (**i**) – Picrosirius Red (polarized light); (**j**,**m**) – Reticulin; (**k**,**n**) – Verhoeff. Scale bars: (**a**) 1 mm; (**g**) 0.2 mm; (**i**) 1000 μm; (**c**) 200 μm; (**d**, **o**) 100 μm; (**b**, **h**, **k**, **l**, **m**, **n**, **p**) 50 μm; (**j**) 20 μm; (**f**) 10 μm; (**e**) 5 μm.

Measurements (length × width × thickness) of the upper eyelid in the crocodile monitor were 21.39 ± 0.6 mm × 10.31 ± 0.5 mm × 4.9 ± 0.8 mm in the female and 24.07 ± 0.4 mm × 12.04 ± 0.5 mm × 6.86 ± 0.6 mm in the male. In contrast, the lower eyelid, measured in the same way, was 21.39 ± 0.6 mm × 16.74 ± 0.4 mm × 9.35 ± 0.9 mm in the female and 24.07 ± 0.4 mm × 19.14 ± 1.1 mm × 10.08 ± 0.8 mm in the male. Macroscopically, the upper and lower eyelids were composed of an external surface (anterior palpebral surface) and an internal surface (posterior palpebral surface/palpebral conjunctiva) (Fig. 1b–f). The palpebral conjunctiva of the upper eyelid consisted of the marginal zone and ocular zone, whereas the posterior palpebral surface of the lower eyelid comprised the marginal zone, plate zone, and ocular zone (Fig. 2a). The external surface of both eyelids was covered with smooth, glossy, tightly apposed scales arranged regularly in longitudinal and transverse rows. The scales were creamy yellow with predominant brown–black pigmentation (Fig. 1b–d). The scales covering the upper and lower palpebral margins were rectangular with rounded ends and exhibited very intense black–brown pigmentation (Fig. 1e, f). The external surface of the upper eyelid was covered with large oval scales, between which numerous small round scales were present (Fig. 1c). In contrast, the lower eyelid scales showed greater variation in shape, ranging from oval through rhomboidal to irregular (Fig. 1d). The posterior palpebral surface was delicately pigmented. Two lacrimal puncta were observed on the lower palpebral margin (Fig. 1e). In the upper eyelid, the posterior palpebral surface was non-pigmented, light pink, and contained clearly defined conjunctival folds (Fig. 1f).

**Fig. 3:**
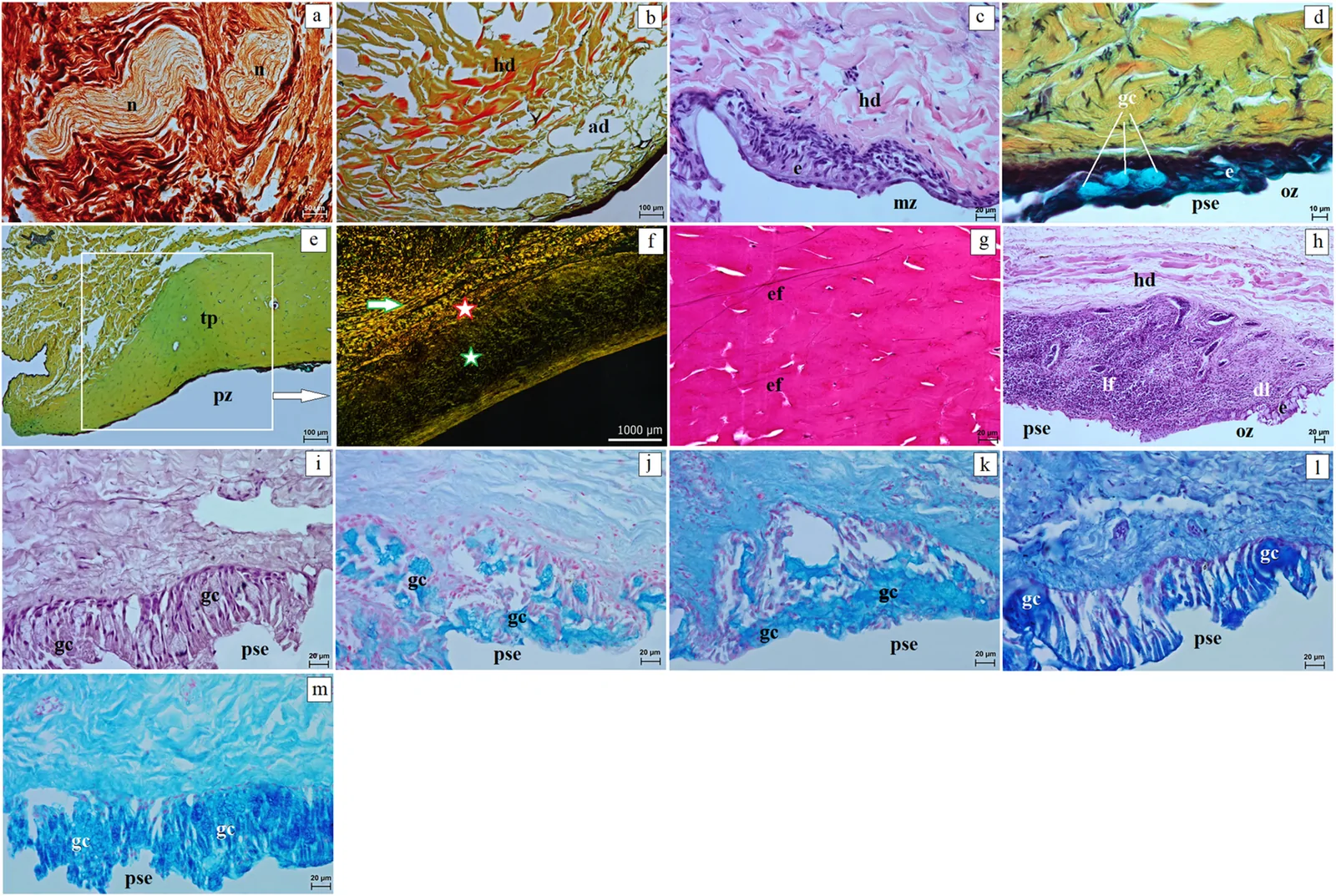
Histology and histochemistry of the upper and lower eyelids in the crocodile monitor. Adipocytes (ad), diffuse lymphocytes (dl), hypodermis (hd), elastic fibers (ef), goblet cells (gc), lymphoid follicle (lf), nerve (n), ocular zone (oz), posterior surface of eyelid (pse), plate zone (pz), tarsal plate (tp); green asterisk and arrow indicate Type III–like collagen (green–yellow birefringence), red asterisk indicates Type I–like collagen (orange–yellow birefringence). (**a**) – Bielschowsky; (**b**,**d**,**e**) – Movat pentachrome (modified Russell–Movat); (**c**,**h**) – Mayer’s H&E; (**f**) – Picrosirius Red (polarized light); (**g**) – Verhoeff; (**i**) – Periodic acid–Schiff (PAS); (**j**) – Alcian blue (AB) pH 1.0; (**k**) – AB pH 2.5; (**l**) – AB pH 2.5/PAS; (**m**) – Colloidal iron (CI). Scale bars: (**k**) 1000 μm; (**b**, **e**) 100 μm; (**a**, **c**) 50 μm; (**c**, **g**–**m**) 20 μm; (**d**) 10 μm.

Histologically, the upper and lower eyelids consisted of the epidermis, dermis, and hypodermis (Fig. 2b–d). The epidermis comprised a superficial cuticle and an outer corneous β-protein (CBP)-rich layer (labeled as the “outer β-keratin layer”). Melanophore pigmentation was present. This was followed by an intermediate fibrous stratum corneum that stained white-red with Picro-Mallory trichrome and contained thick and fine interwoven fibers (likely soft α-keratin), consistent with a flexible hinge region. Beneath the stratum corneum, an inner CBP-rich layer (labeled as the “inner β-keratin layer”) stained intensely dark pink, indicating a more compact and densely organized arrangement of CBPs (formerly termed β-keratins). Melanophore pigmentation was also present in this layer. A thin α-keratin layer and the stratum germinativum (basal layer), which gives rise to the overlying epidermal layers, were located deeper (Fig. 2e). The marginal zone of both the upper and lower eyelids exhibited the highest degree of epidermal pigmentation (Fig. 2f). The next layer of the upper and lower eyelids was the dermis. Adipose tissue was observed directly beneath the epidermis (Fig. 2h). The dermis also contained a thick stratum compactum formed mainly by collagen fibers arranged in parallel (Fig. 2g, h). Polarized light microscopy demonstrated that the dermal stratum compactum showed predominantly green–yellow birefringence, interpreted as being consistent mainly with Type III–like collagen fibers (Fig. 2i). Reticular fibers interwoven among the parallel collagen fibers were also visible (Fig. 2j). Elastic fibers were present as delicate fibrils arranged irregularly relative to the collagen and reticular fibers (Fig. 2k). In both the upper and lower eyelids, an osteoderm was observed. It consisted of woven bone and parallel-fibered bone located between the dermis and hypodermis (Fig. 2a, l). This osteoderm formed a single rectangular plate with rounded edges that integrated with, and coexisted among, numerous scales. The hypodermis was composed of irregularly arranged collagen fibers and contained clearly defined reticular and elastic fibers, nerves, and adipose tissue (Figs. 2m and n and 3a and b). In the upper eyelid, muscle bundles were poorly delineated, whereas in the lower eyelid they were more developed (Fig. 2o, p). The marginal zone of both the upper and lower eyelids was covered by stratified non-keratinized squamous epithelium (approximately 10–11 cell layers in the upper eyelid and 13–16 cell layers in the lower eyelid) (Fig. 3c). The posterior palpebral surface was lined by non-keratinized stratified cuboidal epithelium containing numerous goblet cells (Fig. 3d). Movat pentachrome staining showed a strong positive reaction, confirming the presence of acidic mucopolysaccharides and glycosaminoglycans in the secretion produced by goblet cells (Fig. 3d). In the lower eyelid, within the plate zone, the tarsal plate was oval and macroscopically measured 10.82 ± 0.1 mm × 10.51 ± 0.4 mm in the female and 13.74 ± 0.4 mm × 12.53 ± 0.2 mm in the male. The tarsal plate was composed of compact fibrous connective tissue (dense regular connective tissue). It was dominated by regularly arranged parallel collagen fibers interspersed with delicate elastic fibers (Figs. 1g and 3i). Under polarized light microscopy, the collagen fibers within the tarsal plate showed green–yellow birefringence, interpreted as being consistent mainly with Type III–like collagen fibers (Fig. 3f). In addition, weakly marked fibers with orange–yellow birefringence were observed in the connective tissue surrounding the tarsal plate (Fig. 3f). These fibers were interpreted as being consistent with Type I–like collagen fibers. Furthermore, lymphoid follicles and diffuse lymphocytes were noted in the ocular zone of the lower eyelid (Fig. 3h). Histochemical analysis of goblet cells in the conjunctiva of the upper and lower eyelids revealed a negative PAS reaction, suggesting an absence or very low content of neutral mucins. In contrast, strong (+++) staining was obtained with AB at pH 1.0 and 2.5, AB (pH 2.5)/PAS (blue), and CI, indicating that goblet cell secretion is dominated by acidic mucopolysaccharides (Fig. 3i–m).

### The nictitating membrane

**Fig. 4:**
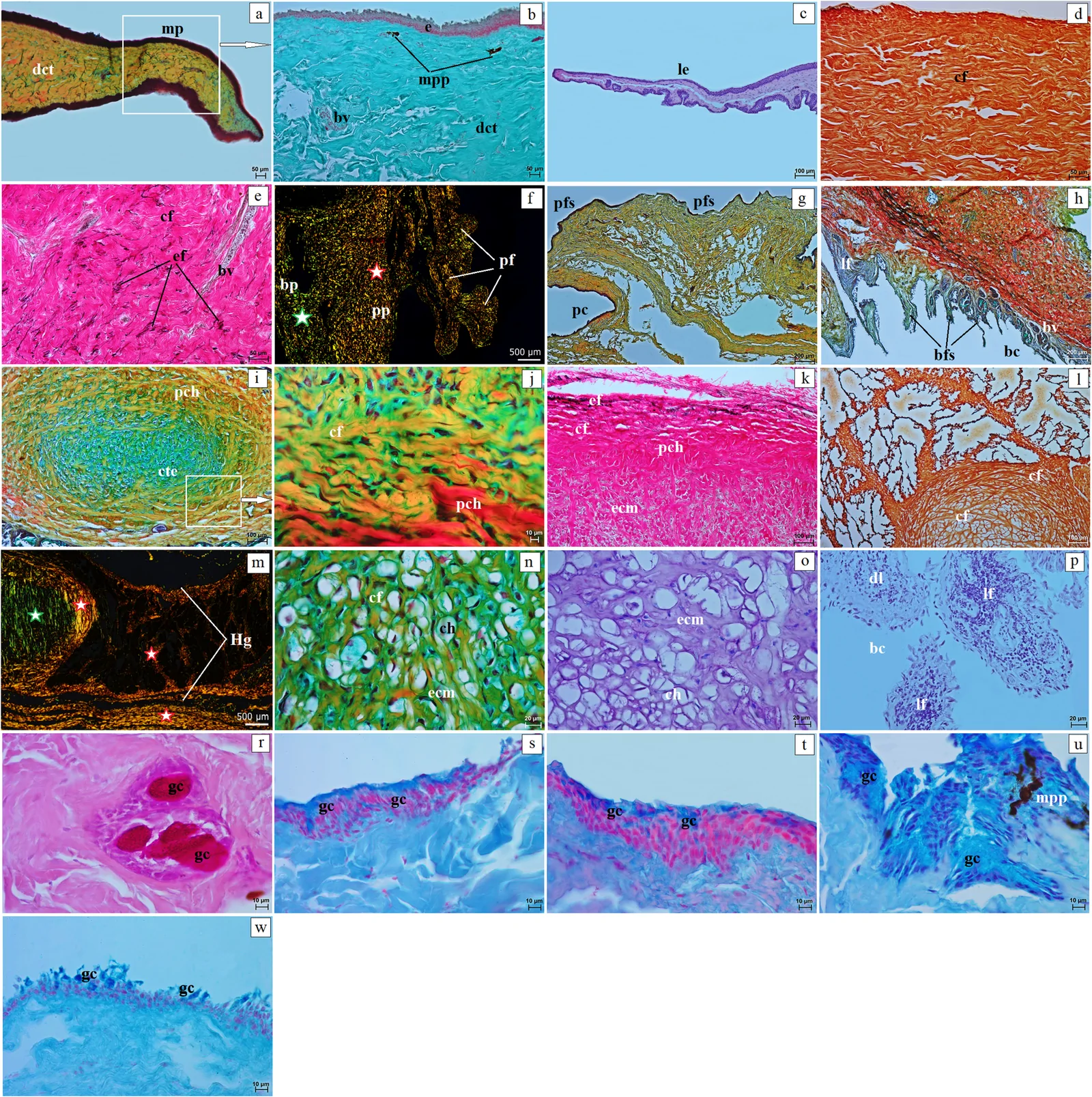
Histology and histochemistry of the nictitating membrane in the crocodile monitor. Bulbar conjunctiva (bc), palpebral conjunctiva (pc), blood vessels (bv), cartilage of the third eyelid (cte), conjunctival folds: bulbar (bfs) and palpebral (pfs), collagen fibers (cf.), diffuse lymphocytes (dl), elastic fibers (ef), dense connective tissue (dct), epithelium (e), extracellular matrix (ecm), goblet cells (gc), Harderian gland (Hg), leading edge (le), lymphoid follicle (lf), marginal plait (mp), melanophore pigmentation (mpp), perichondrium (pch); green asterisk indicates Type III–like collagen (green–yellow birefringence), red asterisk indicates Type I–like collagen (orange–yellow birefringence). (**a**,**g**–**j**,**n**) – Movat pentachrome (modified Russell–Movat); (**b**) – Masson–Goldner trichrome; (**c**,**p**) – Mayer’s H&E; (**d**,**l**) – Reticulin; (**e**,**k**) – Verhoeff; (**f**,**m**) – Picrosirius Red (polarized light); (**o**) – Toluidine blue; (**r**) – Periodic acid–Schiff (PAS); (**s**) – Alcian blue (AB) pH 1.0; (**t**) – AB pH 2.5; (**u**) – AB pH 2.5/PAS; (**w**) – Colloidal iron (CI). Scale bars: (**f**, **m**) 500 μm; (**g**, **h**) 200 μm; (**c**, **i**, **k**, **l**) 100 μm; (**a**, **b**, **d**, **e**) 50 μm; (**n**–**p**) 20 μm; (**j**, **r**–**w**) 10 μm.

Macroscopically, the nictitating membrane of the crocodile monitor consisted of the marginal plait, leading edge, palpebral conjunctiva, and bulbar conjunctiva. Both the palpebral and bulbar conjunctivae contained melanophore pigmentation and exhibited well-developed folds (Fig. 1i–k). The marginal plait and leading edge were lined by non-keratinized stratified squamous epithelium composed of 6–9 to 14–17 layers of nucleated cells (Fig. 4a, c). Directly beneath the epithelium of the marginal plait, small isolated aggregations of melanophores were observed (Fig. 4b). The nictitating membrane stroma was composed of dense regular fibrous connective tissue, consisting mainly of collagen fibers, numerous elastic fiber bundles, and abundant blood vessels (Fig. 4d, e). Polarized light microscopy revealed that the bulbar part of the stroma was predominantly composed of collagen fibers exhibiting green–yellow birefringence. These findings were interpreted as being consistent mainly with Type III–like collagen fibers, whereas the palpebral part primarily contained collagen fibers with orange–yellow birefringence, interpreted as being consistent mainly with Type I–like collagen fibers (Fig. 4f). The palpebral folds of the nictitating membrane contained a mixture of fibers showing orange–yellow and green–yellow birefringence, consistent with a combination of Type I–like and Type III–like collagen fibers (Fig. 4f). The palpebral conjunctiva was lined by non-keratinized stratified cuboidal epithelium, whereas the bulbar conjunctiva was covered by columnar cells. Both palpebral and bulbar conjunctivae formed numerous thick and thin folds containing goblet cells (Fig. 4g, h). The stroma of the nictitating membrane included hyaline cartilage surrounded by a thick perichondrium composed predominantly of collagen fibers, with lightly distributed elastic fibers and numerous fibrocytes (Fig. 4i–l). Under polarized light microscopy, the perichondrium showed densely organized collagen fibers with orange–yellow birefringence, interpreted as being consistent mainly with Type I–like collagen fibers (Fig. 4m). The hyaline cartilage contained chondrocytes arranged in isogenous groups and a small amount of extracellular matrix composed of collagen fibers exhibiting green–yellow birefringence. These fibers were interpreted as being consistent mainly with Type III–like collagen fibers (Fig. 4m, n). Toluidine blue staining revealed metachromasia of the cartilage matrix, indicating high glycosaminoglycan (GAG) content (Fig. 4o). Within the bulbar conjunctiva, numerous lymphoid follicles and diffuse lymphocytes were present (Fig. 4p). Goblet cell secretion was mucous in nature and contained both neutral and acidic mucins. These were demonstrated by strong (+++) PAS, AB pH 2.5, and AB pH 2.5/PAS reactions (blue color), and moderate (++) reactions with AB pH 1.0 and CI staining (Fig. 4r–w).

### The Harderian gland

Macroscopic analysis revealed that the crocodile monitor has two main orbital glands: the Harderian gland and the lacrimal gland. The Harderian gland was a large oval-shaped structure with comparable dimensions between the sexes (Fig. 1l). In the female, the Harderian gland measured 26.77 ± 0.6 mm × 15.94 ± 0.5 mm × 5.86 ± 0.3 mm, and in the male, it measured 28.41 ± 0.6 mm × 16.33 ± 0.4 mm × 5.93 ± 0.2 mm. A noticeable notch on the medial border gave it a kidney-like shape. The Harderian gland was located in the medioposterior region of the eyeball and had a single excretory duct that opened onto the bulbar surface of the nictitating membrane (Fig. 1m).

**Fig. 5:**
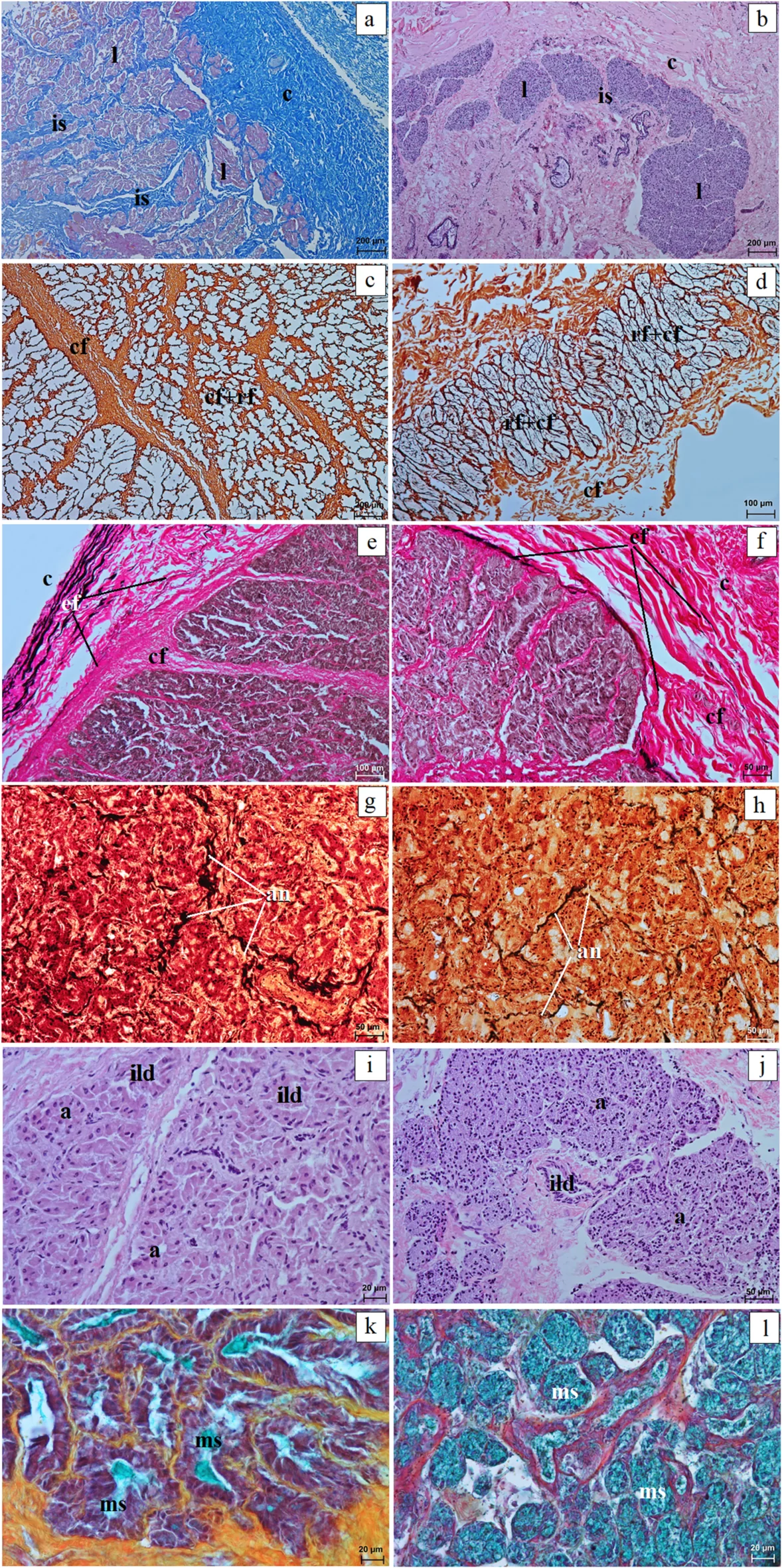
Histology of the Harderian gland (a, c, e, g, i, k) and lacrimal gland (b, d, f, h, j, l) in the crocodile monitor. Acini (a), axonal network (an), capsule (c), collagen fibers (cf.), collagen fibers and reticular fibers (cf. + rf), elastic fibers (ef), intralobular duct (ild), interlobar septa (is), lobes (l), mucous secretion (ms), reticular fibers and collagen fibers (rf + cf.). (**a**) – Heidenhain’s Azan trichrome; (**b**,**i**,**j**) – Mayer’s H&E; (**c**,**d**) – Reticulin; (**e**,**f**) – Verhoeff; (**g**,**h**) – Bielschowsky; (**k**,**l**) – Movat pentachrome (modified Russell–Movat). Scale bars: (**a**, **b**, **c**) 200 μm; (**d**, **e**) 100 μm; (**f**, **g**, **h**, **j**) 50 μm; (**i**, **k**, **l**) 20 μm; (**f**) 10 μm.

**Fig. 6:**
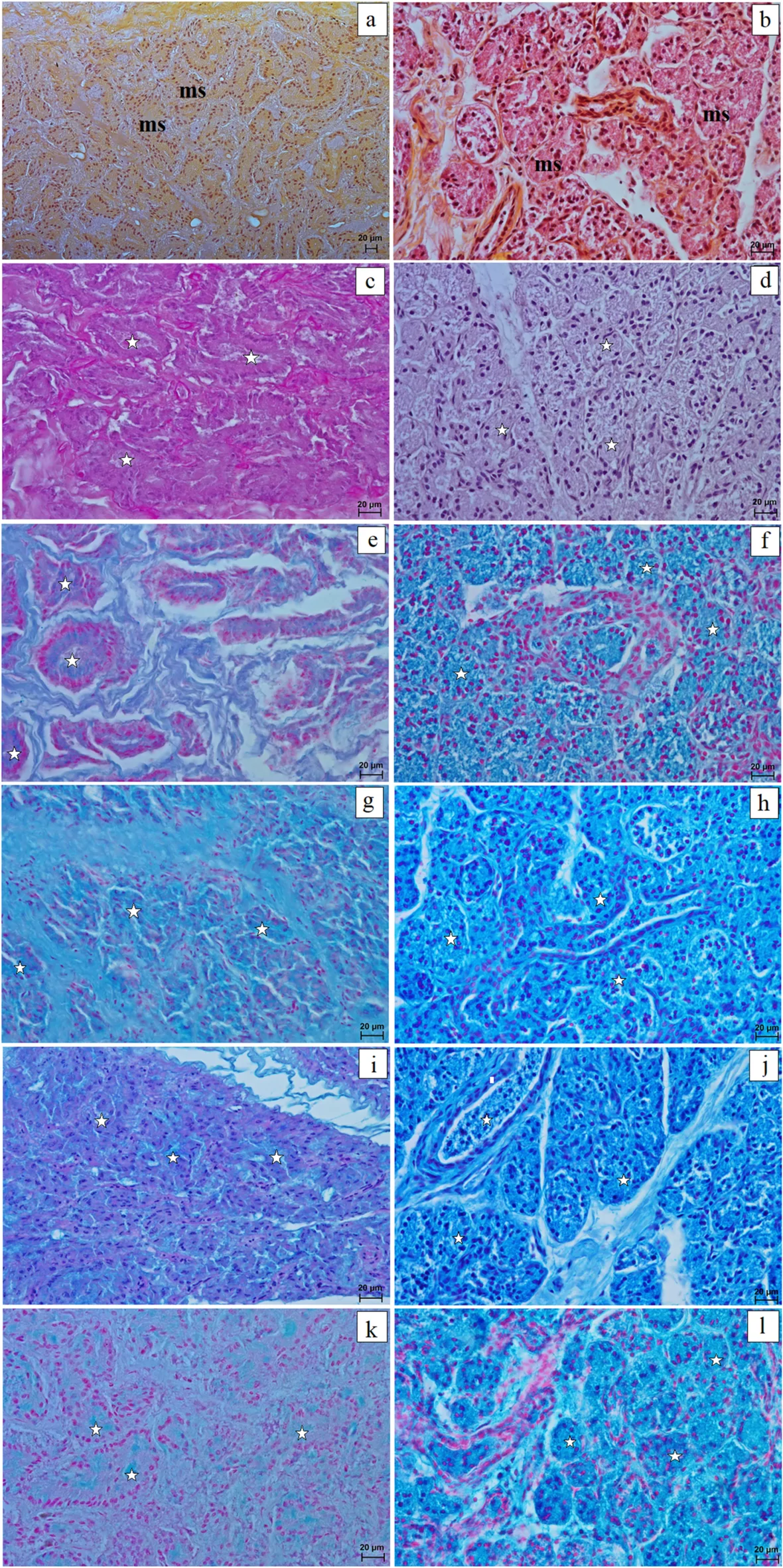
Histochemistry of the Harderian gland (a, c, e, g, i, k) and lacrimal gland (b, d, f, h, j, l) in the crocodile monitor. Mucous secretion (ms); the white asterisk indicates secretory acini. (**a**) – Movat pentachrome (modified Russell–Movat); (**b**) – Mucicarmine; (**c**,**d**) – PAS; (**e**,**f**) – AB pH 1.0; (**g**,**h**) – Alcian blue (AB) pH 2.5; (**i**,**j**) – AB pH 2.5/PAS; (**k**,**l**) – Colloidal iron (CI). Scale bars: (**a**–**l**) 20 μm.

The gland was surrounded by a thin capsule composed of dense irregular connective tissue. The capsule penetrated the gland parenchyma, forming both thick and thin connective tissue septa that divided it into distinct lobes of varying size (Fig. 5a). The capsule consisted primarily of collagen and elastic fibers, with a dense axonal network, whereas reticular fibers were poorly developed (Fig. 5c, e, g). Polarized light microscopy revealed that the connective capsule and interlobar septa were composed of collagen fibers with orange–yellow birefringence, consistent mainly with Type I–like collagen fibers (Fig. 4m). Histologically, the Harderian gland was identified as a compound acinar gland producing mucous secretion. The acini had small lumina and were lined with tall conical cells with eosinophilic cytoplasm and large, round, basally located nuclei. The acini were surrounded by myoepithelial cells. The intercalated ducts were lined with simple columnar epithelium. Numerous interlobular ducts were observed in the central regions of the lobes and within the interlobar septa. These ducts were lined with simple columnar epithelium (Fig. 5i). No histological differences were found between the sexes. Movat pentachrome staining revealed mucous secretory units with moderate (++), green-colored positive reactions, indicating the presence of acidic mucopolysaccharides and glycosaminoglycans (GAGs) (Fig. 5k). Mucicarmine staining also demonstrated a moderate (++) reaction, indicating the presence of neutral mucins (pale yellow coloration) (Fig. 6a). The secretion of the Harderian gland was a mixture of predominantly acidic mucins, mainly carboxylated and sulfated types, as well as moderate amounts of neutral mucins. PAS staining showed a moderate (++) positive reaction, AB pH 1.0 indicated a weak (+) reaction, AB pH 2.5 revealed a strong (+++) reaction, and AB pH 2.5/PAS staining demonstrated a (++/+++) positive reaction (blue coloration). CI staining revealed a +/++ reaction (Fig. 6c, e, g, i, k).

### The lacrimal gland

The lacrimal gland in the crocodile monitor was relatively small. In the female, it measured 4.41 ± 0.07 mm × 2.97 ± 0.1 mm × 1.58 ± 0.08 mm, whereas in the male, it measured 5.15 ± 0.09 mm × 3.39 ± 0.1 mm × 1.63 ± 0.07 mm. The gland appeared light pink and had an elongated shape (Fig. 1n). It was located dorsolaterally to the eyeball. The lacrimal gland was surrounded by a thick capsule composed of irregular dense connective tissue, mainly collagen fibers with sparse elastic fibers (Fig. 5b, d, f). The capsule extended into the gland parenchyma, forming thin connective tissue septa that divided it into large lobes (Fig. 5b). The septa contained numerous reticular fibers, scattered elastic fibers, fibrocytes, and a dense axonal network (Fig. 5h).

Histologically, the lacrimal gland was a compound acinar gland of mucous character. The acini consisted of tall conical cells with narrow lumina, basophilic cytoplasm, and large, round, basally located nuclei. The secretory units were surrounded by myoepithelial cells. The interlobular ducts were lined with simple columnar epithelium, featuring centrally located oval nuclei (Fig. 5j). No histological differences were observed between male and female individuals. Histochemical analysis using Movat pentachrome staining revealed a strong (+++) positive reaction (blue coloration), indicating the presence of acidic mucopolysaccharides and GAGs (Fig. 5l). Mucicarmine staining also showed a strong (+++) reaction, indicating acidic mucopolysaccharides (pink to red) (Fig. 6b). PAS staining was negative, suggesting the absence of neutral glycoproteins and polysaccharides. AB pH 1.0 staining revealed a strong (+++) positive reaction, indicative of sulfated mucins, and AB pH 2.5 also gave a strong (+++) reaction, indicating sialylated and carboxylated acidic mucins. AB pH 2.5/PAS staining showed strong (+++) positivity, with intense blue coloration representing acidic mucins. CI staining indicated moderate (++) to strong (+++) positivity, confirming the presence of sulfated and carboxylated acidic mucins (Fig. 6d, f, h, j, l).

## Discussion

In the crocodile monitor, the eyelid apparatus consists of the upper and lower eyelids and a well-developed nictitating membrane, which provides mechanical protection to the eyeball and supports lubrication and clearing of the corneal surface^20^. The external surface of both eyelids is covered with scales, as reported in multiple reptile clades^13^. The upper eyelid is smaller and less mobile, whereas the lower eyelid is larger and more mobile, consistent with patterns described in the tuatara and several squamate lineages with movable eyelids^33–37^. In species with functional eyelids, two lacrimal puncta are typically present on the posterior surface of the lower eyelid, whereas in snakes, a single, very small punctum is reported^38–40^. Similar findings were observed in the crocodile monitor.

Across Squamata, eyelid morphology varies widely and correlates with lifestyle, ranging from fully movable eyelids to eyelid fusion with a transparent “spectacle”^9,10,13,36,37,41,42^. True movable eyelids occur in some geckos (Eublepharidae), whereas many other geckos lack eyelids and instead have a spectacle^13,37,41^. Similar fused coverings are reported in other lizard groups and may be associated with a reduced or absent nictitating membrane^10,13,36,37^. In snakes, the spectacle is continuous with the periocular skin and encloses a subspectacular space filled by tear film^9,13,42^. These strategies indicate that squamates achieve ocular protection either through movable eyelids and a nictitating membrane or through eyelid fusion and a spectacle. These morphological variations depend on ecological and behavioral demands^13^. Their functional significance should be addressed in further studies.

The morphological features observed during gross anatomical analysis were accompanied by specific microstructural features. Histologically, the upper and lower eyelids show a specialized epidermis with corneous β-protein–rich layers and an α-keratin hinge region. These features may facilitate movement without cracking rigid corneous layers^43^. The fibrous intermediate layer (soft α-keratin) can act as an elastic hinge and shock absorber, supporting rapid eyelid closure/opening and smooth distribution of the tear film^43^. Strong pigmentation of the eyelid margins may additionally reduce UV exposure in the palpebral fissure region. The dermis is compact and reinforced by densely arranged collagen bundles. Similarly, a predominance of Type III–like collagen fibers and the presence of reticular and elastic fibers may support mechanical resilience and flexibility^44,45^. Comparable reinforcement is reported in other reptiles, where organized collagen increases resistance to tearing^45^.

An additional protective component was the presence of osteoderms, which arise in the dermis and represent true bony structures distinct from the keratinized scales^46,47^. Periocular osteoderms have been documented in selected reptile taxa^20,48^. In the lower eyelid, a tarsal plate provides additional support while maintaining flexibility. Across Sauropsida, similar functional demands may be achieved with different tissue architectures^13,33,37^. These findings support the hypothesis that ocular adnexal structures diversify among reptiles in response to environmental adaptation.

The posterior palpebral surface contains numerous goblet cells, and their secretion is important for ocular lubrication and stabilization of the tear film^13^. In many reptiles, goblet cells and the Harderian gland contribute substantially to tear film maintenance, particularly when the lacrimal gland is small or reduced^13,49^. Because the ocular adnexa play a crucial role in eye protection, reduction of one accessory ocular structure may be compensated by an increased functional contribution of other structures.

The lymphoid tissue in the palpebral conjunctiva is also consistent with local immune protection of the ocular surface. As part of the mucosa-associated lymphoid tissue^50^, it is comparable to other reports from reptiles and birds^51–53^. Thus, normal function of this tissue likely plays an important role in immune defense of the eye and in preventing infections visible during clinical examinations. CALT deficiency may be an important clinical problem in exotic animals, especially when individuals are maintained in artificial environments.

The nictitating membrane contains hyaline cartilage and conjunctiva-associated lymphoid tissue. Thus, it combines both mechanical and immune defense of the eye. Collagen architecture of the cartilage support contributes to tensile strength and mechanical stability^54,55^, whereas Type III–like collagen fibers may support flexibility and resilience^32,55,56^. The presence of cartilage within the nictitating membrane has been interpreted as a specialized feature linked to increased mechanical protection in some taxa^57–59^. Glycosaminoglycans in the cartilage matrix confer hydrophilic properties and resistance to compression^44^, supporting the structural role of the nictitating membrane. Cartilage within the nictitating membrane has also been reported in the tuatara^37^. Moreover, cartilaginous elements and the bursalis muscle tendon have been described in several lizard clades. These findings suggest the potential for active movement comparable to the mechanism described in birds^60^. In crocodilians and birds, rigidity of the nictitating membrane has been linked to an organized network of collagen fibers^52,61^, although some authors also indicate cartilaginous support in non-burrowing forms^11,12,62^. Variation in pigmentation and vascularization can influence nictitating membrane transparency (e.g., in *Caiman latirostris*)^61^, and melanophore presence may reduce transparency in the crocodile monitor. The nictitating membrane is present in some geckos (e.g., *Coleonyx*), whereas it is absent from chameleons and some pygopodids^23,38,63^. The immunological and secretory aspects of the nictitating membrane are also relevant. CALT provides a first line of defense^52^, and mucus produced by goblet cells stabilizes the tear film^51^. In birds, the tear film may additionally include lipids from the Harderian gland, reducing evaporation^49,64^. In *Dermochelys coriacea*, the folded conjunctiva of the nictitating membrane increases surface area for mucus production and may protect the cornea from highly concentrated salt gland secretions^65^. In the crocodile monitor, folded conjunctiva may similarly support ocular surface protection in a humid, densely vegetated environment. Overall, these observations describe the complex and divergent role of the nictitating membrane in ocular homeostasis.

The secretion of the ocular glands represents another aspect of eye defense. The orbital glands are represented by the Harderian gland and the lacrimal gland. As in many squamates with movable eyelids, the Harderian gland is the dominant gland closely associated with the nictitating membrane. The lacrimal gland is smaller and located dorsolaterally^37,66,67^. The Harderian gland drains onto the bulbar surface of the nictitating membrane, indicating a direct contribution to the tear film. In this rainforest species, predominantly mucous secretion can be interpreted primarily as supporting hydration, lubrication, and removal of fine debris encountered during movement through dense vegetation^8,9,13^. This function seems especially important from an environmental-adaptive perspective. The Harderian gland shows marked interspecific variability in structure and secretion type across Squamata^13,16,17,23,37,66–68^. This variability has also been linked to eyelid anatomy and ecological demands. A chemosensory role of Harderian gland secretion is discussed for some reptiles, particularly snakes^66,69^. In the crocodile monitor, the topography and mucous secretion are most consistent with a primary role in ocular surface homeostasis. Simultaneously, the lacrimal gland remains a smaller accessory gland and, together with goblet cells, likely complements the Harderian gland in maintaining the tear film^14,37,66–68^. The dominant Harderian gland and other exocrine units (lacrimal gland and goblet cells) form the tear film, which, together with mechanical defense (eyelids) and immune structures (CALT), allows efficient protection of vision in both natural and captive environments.

## Conclusions

In the crocodile monitor, the upper eyelid, lower eyelid, nictitating membrane, and orbital glands form a protective system that provides mechanical protection and supports ocular surface homeostasis. The upper and lower eyelids are scale-covered and show a CBP-rich epidermis with a collagen-reinforced hinge region. The osteoderms provide additional reinforcement. The posterior palpebral surfaces contain abundant goblet cells producing mainly acidic mucins, and the lower eyelid includes a supportive fibrous tarsal plate and lymphoid tissue. The nictitating membrane contains hyaline cartilage with a glycosaminoglycan-rich matrix and CALT. The Harderian gland is the dominant orbital gland, drains onto the bulbar conjunctiva of the nictitating membrane, and produces mainly acidic mucins with a moderate neutral component. The smaller dorsolateral lacrimal gland produces predominantly acidic mucins. In summary, this study describes the first complete morphological and secretory characteristics of the ocular adnexa in the crocodile monitor. These findings provide a reference for future morphological and clinical studies of the eye in crocodile monitors.

## Limitations

The analyses were performed on material obtained from two crocodile monitors from a zoological garden. Therefore, it cannot be excluded that some functionally relevant features, particularly those related to secretory activity and histochemical profiles, may have been influenced by living conditions, and the results should be interpreted in this context. Although Picrosirius Red staining under polarized light is useful for assessing collagen organization, birefringence color is influenced by fiber thickness, packing, and section orientation. Thus, collagen type assignment (e.g., Type I–like collagen vs. Type III–like collagen) should be considered tentative unless confirmed by collagen-specific methods.

### Future recommendations

Future studies should include a larger number of individuals, ideally from multiple zoological institutions or rehabilitation centers, to assess intraspecific variation and the potential influence of artificially controlled environments on ocular adnexal tissues. When available, cadavers of wild-living individuals, as well as comparative data from museum collections, would help determine species-typical features. Combining morphological findings with clinical ophthalmic assessment, including evaluation of the ocular surface and tear film, may better link eyelid and gland structure with protective function. The collagen composition in key supportive structures (the upper and lower eyelids, the tarsal plate, and the nictitating membrane cartilage/perichondrium) should be confirmed using collagen-specific approaches, such as immunohistochemistry or immunofluorescence for Type I–like collagen and Type III–like collagen, and, where feasible, in situ hybridization for COL1A1 and COL3A1, complementing polarized light microscopy.

## Supplementary Information

Below is the link to the electronic supplementary material.

Supplementary Material 1

## Data Availability

The datasets generated during and/or analyzed during the current study are available from the corresponding author on reasonable request.
