# Supplementary material for: Morphological and histochemical characterization of eyelids and orbital glands in the crocodile monitor (Varanus salvadorii)
Source: Sci Rep. 2026 Jun 11;16:26575. doi: 10.1038/s41598-026-57505-2 (PMC13503834; doi:10.1038/s41598-026-57505-2)
Supplement: Supplementary file 1 [file 41598_2026_57505_MOESM1_ESM.docx]

Supplementary file

Tissue processing and paraffin embedding:

1. the tissue samples fixation in 4% buffered formaldehyde (Chempur, Piekary Śląskie, Poland) for at least 72 hours,
2. the tissue samples rinsing in the running water for 24 hours,
3. the tissue dehydration using the descending grade of ethanol solutions (75%, 96%, and 100%) (Chempur, Piekary Śląskie, Poland) in a vacuum tissue processor – ETP (RVG3, Intelsint, Italy), followed by paraffin embedding.

Histological slides preparation:

1. Tissue sections of 4 μm thickness with a sliding microtome (Slide 2003, Pfm A.G., Germany).

Microscopic imaging:

1. Zeiss Axio Scope A1 light microscope (Carl Zeiss, Jena, Germany) equipped with Axio Vision Release 4.8.2 SP 2 software.

Histochemical techniques:

1. The results were interpreted using the subsequent scale: (–) – no reaction; (–/+) and (+) – weakly positive reaction; (++) and (++/+++) – moderately positive reaction; (+++) – strongly positive reaction.

All procedures involving formaldehyde (Chempur, Piekary Śląskie, Poland), xylene (Chempur, Piekary Śląskie, Poland), and picric acid (Chempur, Piekary Śląskie, Poland) were performed in a fume hood with appropriate personal protective equipment, following institutional safety regulations.

Histological and histochemical staining:

1. Mayer’s hematoxylin and eosin (Sigma-Aldrich, Taufkirchen, Germany) (nuclei – dark blue color; cytoplasm and extracellular matrix proteins – pink color),
2. Heidenhain’s Azan trichrome (Bio-Opitca Milano S.p.A., Milan, Italy; cat. no. 04-001802) (collagen fibers and mucus – blue color),
3. Masson–Goldner trichrome (Bio-Opitca Milano S.p.A., Milan, Italy; cat. no. 04-011802) (collagen fibers and mucus – green color; keratin and muscle fibers – red color),
4. Mallory trichrome with aniline blue (Bio-Optica Milano S.p.A., Milan, Italy; cat. no. 04-020802) (collagen fibers and mucus – blue color; keratin and muscle fibers – red color),
5. Reticulin (BioGnost Ltd., Zagreb, Croatia; cat. no. RE-100T) (reticulin fibers – dark violet to black); and Verhoeff (BioGnost Ltd., Zagreb, Croatia; cat. no. VER-100T) (elastic fibers – black),
6. Movat pentachrome (modified Russell–Movat) (BioGnost Ltd., Zagreb, Croatia; cat. no. MOV-100T) for mucin secretion detection (acid mucopolysaccharides and glycosaminoglycans (GAGs) – blue to green),
7. Mucicarmine (Bio-Optica Milano S.p.A., Milan, Italy; cat. no. 04-190812) (acid mucins – various shades of red; neutral mucins – pale yellow),

Specialized components identification:

1. Bielschowsky (BioGnost Ltd., Zagreb, Croatia; cat. no. BLS-100T) (axons and neurofibrils – dark brown to black),
2. Masson–Fontana (BioGnost Ltd., Zagreb, Croatia; cat. no. MF-100T) (melanin granules – black),
3. Toluidine blue (Bio-Optica Milano S.p.A., Milan, Italy; cat. no. 05-B23001) (cartilage – metachromasia: violet, purple or pink-violet).

Glycans and mucosubstances characterization:

1. Periodic acid–Schiff (PAS) (Bio-Optica Milano S.p.A., Milan, Italy; cat. no. 04-130802) reaction in glycans, glycoproteins, and neutral mucins visualization,
2. Alcian blue (AB) pH 1.0 (Bio-Optica Milano S.p.A., Milan, Italy; cat. no. 04-166802) in strongly sulfated GAGs visualisation,
3. Alcian blue (AB) pH 2.5 (Bio-Optica Milano S.p.A., Milan, Italy; cat. no. 04-160802) in acidic sialylated GAGs visualization,
4. Alcian blue (AB) pH 2.5/PAS (Bio-Optica Milano S.p.A., Milan, Italy; cat. no. 04-163802) in sulfated and carboxylated acid mucopolysaccharides, as well as sulfated and carboxylated sialomucins (glycoproteins) (blue color) and neutral mucins (magenta color) identification,
5. Colloidal iron (CI) (Bio-Optica Milano S.p.A., Milan, Italy; cat. no. 04-180809) in presence of sulfated acid mucosubstances (SAM) and carboxylated acid mucosubstances (CAM) detection.

Polarized light microscopy:

1. Picrosirius Red a commercial kit (Abcam, Cambridge, UK, cat. no. ab150681), following the manufacturer’s protocol:
   a. deparaffinized and rehydrated tissue sections stained in Weigert’s hematoxylin (Sigma-Aldrich, Taufkirchen, Germany) for 8 minutes to visualize cell nuclei,
   b. rinsing with tap water for 5 minutes,
   c. after staining, slides washing according to the kit protocol: dehydrated through graded ethanol solutions (70%, 95%, and 100%) (Chempur, Piekary Śląskie, Poland), cleared in xylene (Chempur, Piekary Śląskie, Poland), and mounted using a resin-based DPX medium (Sigma-Aldrich, Taufkirchen, Germany) [31].
2. The stained sections examination under a polarized-light microscope (Delta Optical sp. z o.o. sp. k., Nowe Osiny, Poland) to differentiate collagen types:
   a. thick collagen fibers – primarily Type I–like collagen – appeared orange-yellow,
   b. thinner fibers – mainly Type III–like collagen – appeared green-yellow.
3. Usage of ×4 magnification, both crossed polarizers and transmission mode.
4. Series of images was stitched using ImageJ (U. S. National Institutes of Health, Bethesda, Maryland, USA) with no additional color correction was applied to the acquired images.
